# Ethylene glycol elimination in amine loop for more efficient gas conditioning

**DOI:** 10.1186/s13065-018-0493-3

**Published:** 2018-11-23

**Authors:** Nasibeh Hajilary, Mashallah Rezakazemi

**Affiliations:** 1 Department of Chemical Engineering, Faculty of Engineering, Golestan University, Gorgan, Iran; 2 Faculty of Chemical and Materials Engineering, Shahrood University of Technology, Shahrood, Iran

**Keywords:** CO_2_ and H_2_S absorptions, Mono ethylene glycol, Amine gas sweetening, Corrosion, Foaming

## Abstract

The gas sweetening unit of phase 2 and 3 in South Pars Gas Field (Asalouyeh, Iran) was first simulated to investigate the effect of mono ethylene glycol (MEG) in the amine loop. MEG is commonly injected into the system to avoid hydrate formation while a few amounts of MEG is usually transferred to amine gas sweetening plant. This paper aims to address the points where MEG has negative effects on gas sweetening process and what the practical ways to reduce its effect are. The results showed that in the presence of 25% of MEG in amine loop, H_2_S absorption from the sour gas was increased from 1.09 to 3.78 ppm. Also, the reboiler temperature of the regenerator (from 129 to 135 °C), amine degradation and required steam and consequently corrosion (1.10 to 17.20 mpy) were increased. The energy consumption and the amount of amine make-up increase with increasing MEG loading in amine loop. In addition, due to increasing benzene, toluene, ethylbenzene and xylene (BTEX) and heavy hydrocarbon solubility in amine solution, foaming problems were observed. Furthermore, side effects of MEG presence in sulfur recovery unit (SRU) such as more transferring BTEX to SRU and catalyst deactivation were also investigated. The use of total and/or partial fresh MDEA, install insulation and coating on the area with the high potential of corrosion, optimization of operational parameters and reduction of MEG from the source were carried out to solve the problem. The simulated results were in good agreement with industrial findings. From the simulation, it was found that the problem issued by MEG has less effect when MEG concentration in lean amine loop was kept less than 15% (as such observed in the industrial plant). Furthermore, the allowable limit, source and effects of each contaminant in amine gas sweetening were illustrated.

## Introduction

Natural gas is produced from wells with a range of impurities and contaminants such as sulfur dioxide (SO_2_), hydrogen sulfide (H_2_S) and carbon dioxide (CO_2_) [1–4]. These contaminants should be removed from the natural gas to meet typical specifications for use as commercial fuel or feedstock for natural gas hydrate, liquefied natural gas (LNG) plants, gas turbines, industrial and domestic use [5–8]. Removal of these contaminants is required from point of safety, environmental requirements, corrosion control, product specification, decreasing costs, and prevention of catalysts poisoning in downstream facilities [9].

Many methods have been employed to remove acidic components (primarily H_2_S and CO_2_) from hydrocarbon streams including adsorption, absorption [10, 11], membrane [12–16], hybrid system and etc. [17–20]. From these methods, the amine absorption attracts increasing attention due to higher H_2_S and CO_2_ removal and environmental compliance. An amine gas treating plant is commonly faced with two major problems: corrosion and instability of operation [6]. Furthermore, the purity of amine has a considerable effect on the efficiency of the gas sweetening unit. In most amine based sour gas treating process, the conventional alkanol amines such as monoethanolamine (MEA), diethanolamine (DEA), methyl diethanolamine (MDEA), disopropanolamine (DIPA), and diglycolamine (DGA) is used to separate H_2_S and CO_2_ from natural gas [19, 21]. MDEA is commonly used in industrial plants because it has some advantages over other alkanol amines such as high selectivity to the H_2_S, high equilibrium loading capacity (1 mol CO_2_ per 1 mol amine) and less heat of reaction with CO_2_, and lower energy consumption in regeneration section.

Mono ethylene glycol (MEG) is commonly injected into the system from two different points (wellhead and gas receiving facilities) as corrosion and hydrate inhibitor especially during winter time when the potential of condensation corrosion and hydrate formation are high. In phases 2 and 3 through the gas path, MEG is injected at sea line, before HIPPS valve, and after the High-pressure separator drum. A few amounts of MEG is usually transferred to the amine gas sweetening plant. The MEG concentration gradually increases in amine gas sweetening plant even to more than 25%. A large build-up of injection chemicals can eventually lead to fouling and can cause changes in solution physical properties, such as viscosity and mass transfer.

South Pars is a giant gas reservoir shared with Qatar with more than 20 phases. The phases 2 and 3 of South Pars gas refinery has been planted to treat the produced gas through four gas treating trains and stabilize the accompanied condensate from the gas reservoir. Nowadays, about 2500 million standard cubic feet per day (MMSCFD) of gas is fed to this plant. In phases 2 and 3, the untreated gas is transferred via two 30″ pipelines to onshore facilities for treatment. MEG is transferred by means of two 4″ piggy back lines to the wellhead for hydrate prevention and low dosage hydrate inhibitor (LDHI) is being used as a backup.

The main purpose of the current study is to find where MEG has negative effects on gas sweetening process and what the practical ways to reduce its effect are. The effects of MEG injection on amine gas sweetening and sulfur recovery unit (SRU) units were also studied. Since the presence of MEG was not predicted in the design of gas sweetening unit, it seems the phases 2 and 3 was the first gas plants to deal with this problem. Other gas refineries in South Pars Gas Field which used MEG as a hydrate inhibitor are gradually encountering this problem. Furthermore, a certain value was not found in the literature for the maximum allowable of MEG content in amine loop. To overcome the problems issued by MEG in amine loop, four different methods including: (1) changing operational parameters in the presence of MEG in amine loop; (2) reducing MEG loading in amine loop by total or partial discharging of amine; (3) enhancing resistant to corrosion; (4) developing a strategy to track the source of MEG in amine loop were suggested and investigated.

## Gas sweetening unit description

Phases 2 and 3 of South Pars Gas Field were designed for processing of sour gas by means of four MDEA based amine units (licensed by ELF Aquitaine which does not need to remove all CO_2_; resulting in high H_2_S content in acid gas for Claus SRU). The composition of sour gas feed is reported in Table 1. The sour feed gas contains 0.6% H_2_S and 2% CO_2_.

**Table 1:** Characteristics of sour gas feed to the gas sweetening unit (units 101 and 108) of phases 2 and 3 in South Pars Gas Field (Asalouyeh, Iran)

Components	Mole%
H_2_S	0.5548
CO_2_	1.8303
C_1_	85.1012
C_2_	5.4372
C_3_	1.9888
i-C_4_	0.368
n-C_4_	0.5709
i-C_5_	0.1766
n-C_5_	0.1574
Benzene	0.0194
N_2_	3.4754
*n*-hexane	0.0674
Cyclo hexane	0.0299
Methyl cyclo pentane	0.0195
toluene	0.0046
Methyl cyclo hexane	0.0094
Heptane	0.0604
Octane	0.0324
Ort-xylene	0.0048
Nonane	0.003
Decane	0.0003
Carbonyl sulphide	0.003
Methyl mercaptans	0.0021
Ethyl mercaptans	0.0137
Propyl mercaptans	0.0037
Butyl mercaptans	0.0008
Ort-xylene	0.0048

**Fig. 1:**
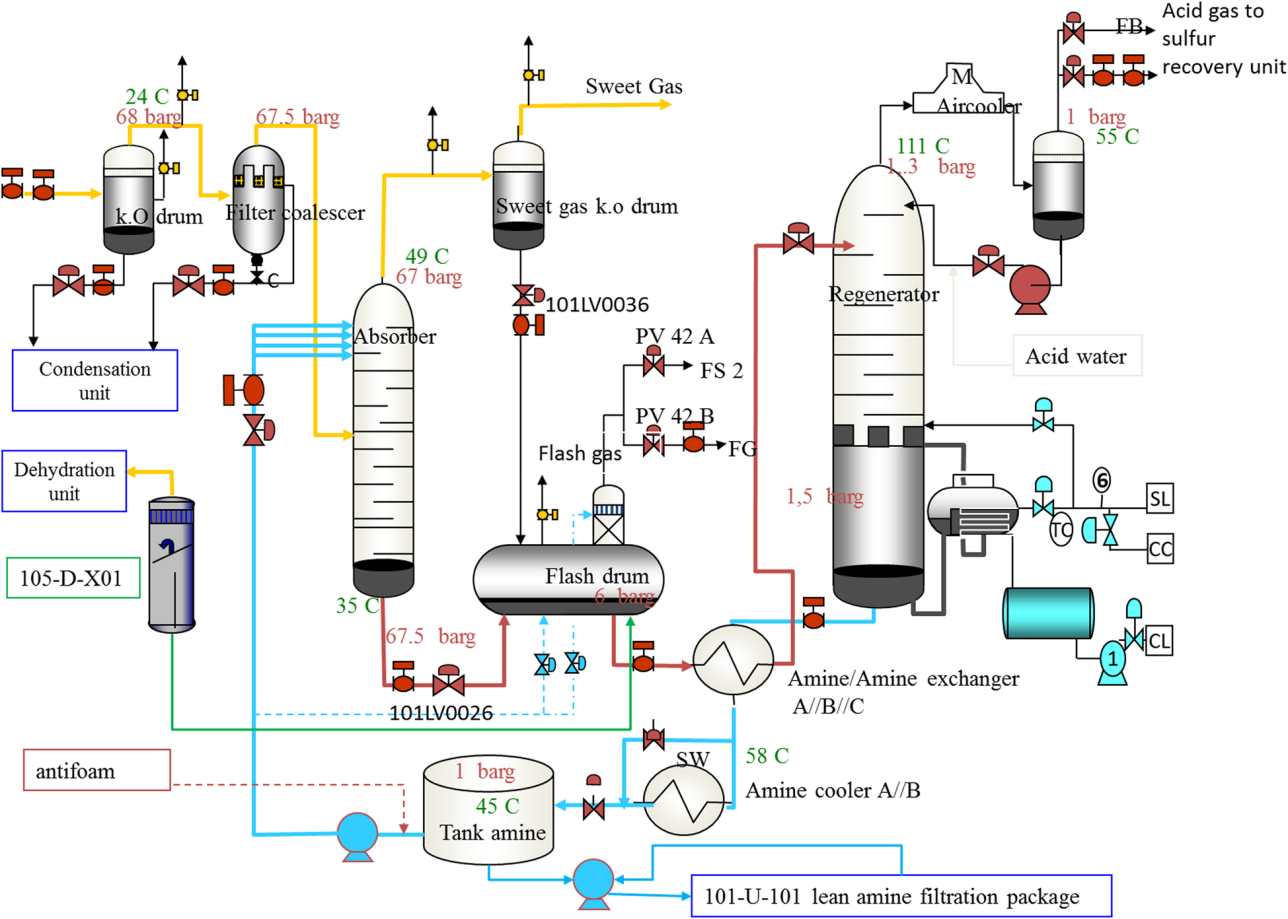
Schematic of the gas sweetening unit (Unit 101) of phases 2 and 3 in South Pars Gas Field (Asalouyeh, Iran) designed by total company

The objective of the gas treatment unit is to meet the design sweet gas specification which must contain less than 4 ppmv H_2_S and 1 mol% CO_2_ and produce suitable acid gas for processing in the SRU’s. This certain specification of product in industrial plants is commonly achieved through an amine unit including absorption and a regeneration sections. In the absorber, amine solution absorbs H_2_S and CO_2_ from the sour gas to produce a sweetened gas stream and a rich amine (a rich amine is an aqueous solution which has absorbed the H_2_S and CO_2_). The rich amine after passing through a flash drum and increasing its temperature in some exchangers routed into the MDEA regenerator (a stripper with a reboiler) to produce lean amine (a lean amine is a solution regenerated from acid gases) that is come back to the absorber. The stripped acid gas from the regenerator with a high concentration of H_2_S (more than 30%) and CO_2_ (less than 60%) is routed into a Claus SRU to produce the liquid sulfur. Sweet gas from the absorber is also routed to the dehydration unit. A schematic of phases 2 and 3 of gas sweetening unit is shown in Fig. 1. Chemical reactions take place in the absorber is shown in Eqs. (1 and 2) and the same but opposite take place in the regenerator.

1 $$MDEA + H_{2} S \to MDEAH^{ + } HS^{ - }$$

2 $$MDEA + H^{ + } HCO_{3}^{ - } \to MDEAH^{ + } HCO_{3}^{ - } .$$

**Fig. 2:**
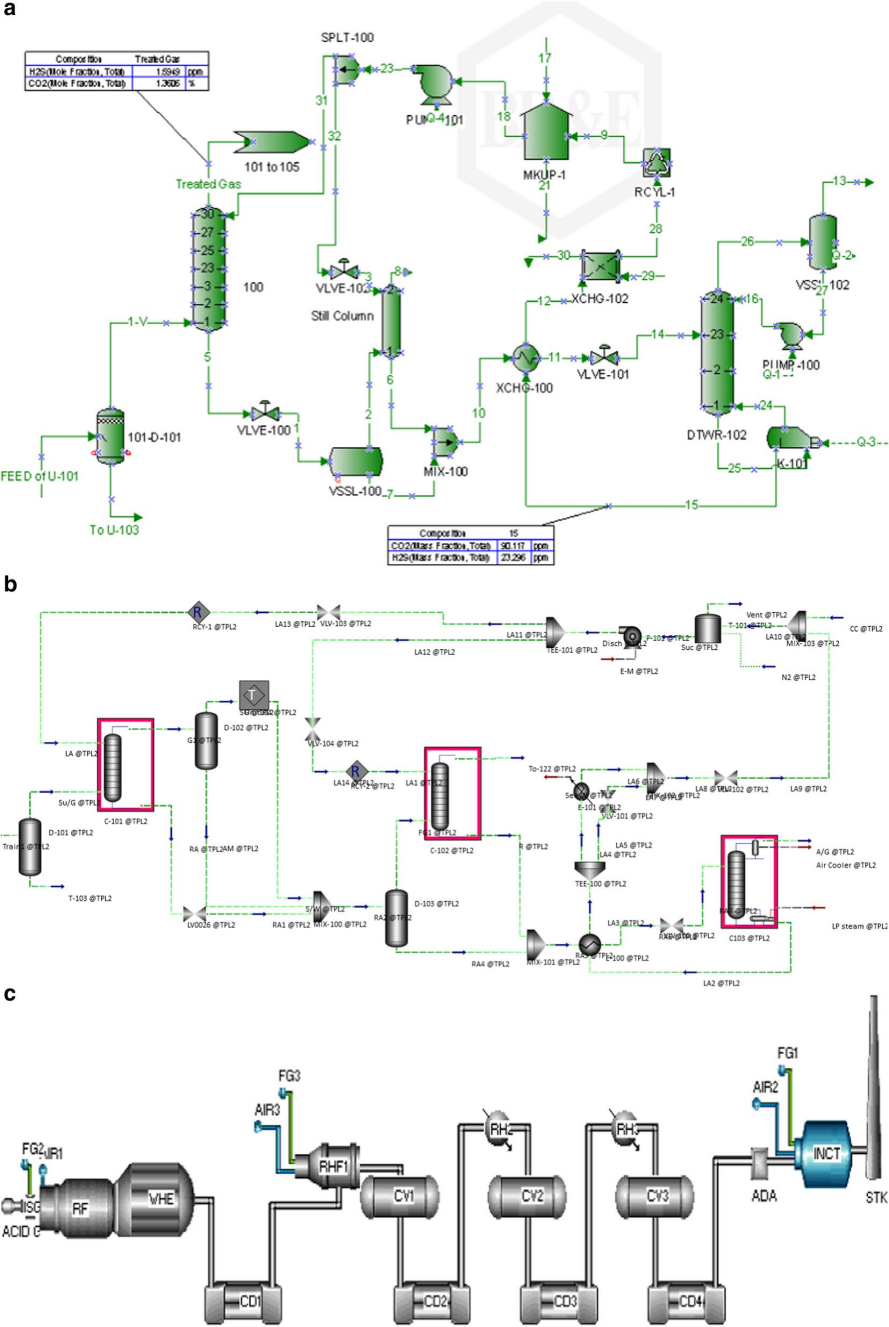
Schematic of the simulated gas sweetening unit [unit 101 of phases 2 and 3 in South Pars Gas Field (Asalouyeh, Iran)] as from **a** ProMax, **b** Aspen HYSYS and **c** SULSIM software

In this research, the gas sweetening and sulfur recovery units (SRUs) (Units 101 and 108, phases 2 and 3, South Pars Gas Field, Asalouyeh, Iran) were simulated using ProMax (Version 2.3) and Aspen HYSYS (version 7.8), and SULSIM (version 6) simulators and a schematic of the simulations are shown in Fig. 2. The process simulations were used to perform a parametric study to predict the operational parameters change as a function of MEG content in amine loop and also to better identifying of operational conditions. Acidic gases and amines are weak electrolytes, which partially dissociate in the aqueous phase. Hence, electrolyte-NRTL model and Soave–Redlich-Kwong (SRK) equation for thermodynamically modeling of state in Aspen HYSYS were used. Also, “amine sweetening PR” property package and “TSWEET” kinetics model were selected in ProMax to provide complete information about ionic analysis, mass, and molar flow of the streams [22]. The simulated results were in good agreement with industrial findings (Table 2). The properties of MEG are reported in Table 3.

**Table 2:** The comparison of the simulation results of the gas sweetening unit with Promax with actual data

Location	Parameters	Simulation results	Actual data
Lean amine	MDEA%	45	45
	MEG%	15	15
	Amine flow rate (m^3^/h)	155	155
Inlet to regenerator	Amine temperature (°C)	102	101.8
Regenerator	Top temperature (°C)	100.2	100.6
	Bottom temperature (°C)	134.39	132.68
Amine inlet to the regenerator reboiler	CO_2_ loading (mol%)	0.018	0.017
	H_2_S loading (mol%)	0.043	0.046
	H_2_S loading mole/mole amine	0.0038	0.0046
	CO_2_ loading mole/mole amine	0.0016	0.0018
Gas in the absorber top	H_2_S (ppm)	1.9	2.02
	CO_2_ (%)	1.3	1.33
Amine in the absorber bottom	CO_2_ loading mole/mole amine	0.11	0.12
	H_2_S loading mole/mole amine	0.21	0.24
	CO_2_ (mol/h)	67.9	67.78
	H_2_S (mol/h)	129.1	129.1

**Table 3:** Chemical properties of MEG

Properties	Value
Molecular weight (g/mol)	62.069
Normal boiling point (°C)	197.248
Ideal liquid density (kg/m^3^)	1110.71
Viscosity @ 60 °C (cP)	5.2
Flash point (°C)	111

## Results and discussion

### Regenerator bottom temperature

**Fig. 3:**
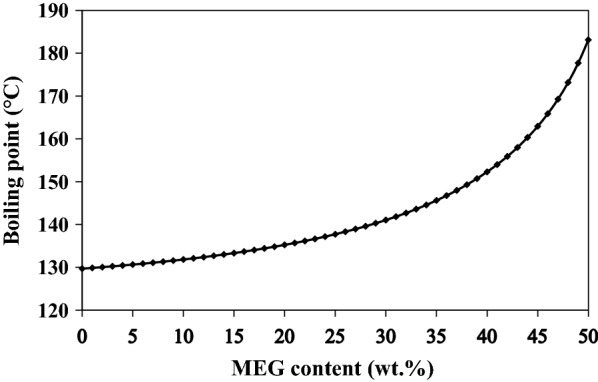
Variation of boiling temperature of lean amine solution containing 45 wt% MEDA as a function of MEG loading

The primary or secondary amines in MDEA solution are commonly formed at higher temperatures because MDEA would go through demethylation/dealkylation process [23]. MEA and DEA are formed by replacing alkyl groups with hydrogen atoms in MDEA using the free radical mechanism. Hence, the effect of the regenerator bottom temperature on amine degradation was investigated. Since the various MEG concentrations affect the boiling point of the solution in the system, the variation of boiling temperature of the aqueous solution of MDEA at a 45 wt% concentration as a function of MEG loading is illustrated in Fig. 3. As can be seen, the boiling point of aqueous MDEA solution increases in presence of MEG content. This boiling point elevation occurs because the boiling point of MEG is higher than that of water, indicating that an MDEA/MEG solution has a higher boiling point than a pure MDEA.

**Fig. 4:**
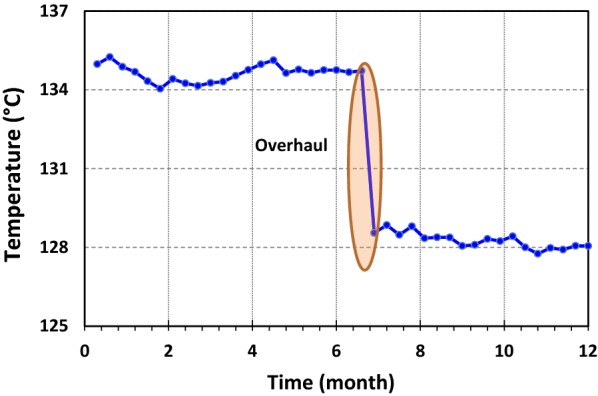
Regenerator bottom temperature in gas sweetening unit. Overhaul: scheduled shutdown maintenance

The primary and secondary amines are commonly not selective to H_2_S and they are more corrosive and need high steam demand for regeneration in compare to MDEA. To prevent primary or secondary amines formation in MDEA solution, the temperature of the reboiler shall not increase more than 132 °C. According to the temperature trends of reboiler (Fig. 4), this value exceeds frequently and after using fresh amine, the reboiler temperature decreases to the allowable range (less than 130 °C). Inducing high temperature degrades amine, produces some acids causing corrosion. Indeed, amine reacts with acids and forms heat stable salts (HSS). This issue may carry out when the stability of salt reduced in the places where some disassociations occur in a site-specific location in the gas sweetening unit. Corrosion takes place when that disassociations form a corrosion cell with metal in the unit. Some issues are also appeared by the chelating effect of the formed acids. The chelating effect is the increased affinity of chelating ligands toward a metal ion in comparison to the affinity of similar non-chelating ligands toward the same ion. However, the chelating effect may keep the iron in the aqueous solution, rather than leading it to create a protective layer on the metal; therefore, acid corrosion occurs and amine degrades [24]. The simulation results also indicated that for the same circulation rate at the same process conditions, when MEG content in amine loop were 0, 5, 15, 0 and 25 wt%, the regenerator bottom temperatures were 129.6, 130.6, 131.8, 133.2, 135.2 and 137.7 °C, respectively. The field data (Fig. 4) confirmed the simulation results.

### H_2_S absorption

**Fig. 5:**
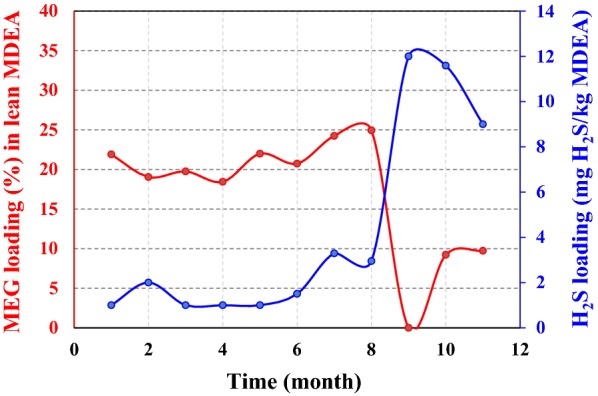
MEG concentration versus acid gas loading in lean amine solution

From screening the results presented in Fig. 5, it can be realized that the maximum acid gas loading (12 mg H_2_S/kg MDEA) occurs at the minimum MEG concentration (0 wt%). Actually, the zero value of MEG concentration indicates the used lean amine has become discharged from the tank and the fresh amine is loaded into the tank. In a case, from the field data, the reboiler temperature was 128 °C with MEG concentrations of 10 wt% in gas treating trains #1 and #2 while in trains #3 and #4, the reboiler temperature was 133 °C with 20 wt% MEG concentration. As mentioned, to prevent primary or secondary amines formation in MDEA solution, the reboiler temperature shall not exceed 132 °C [24]. As can be seen, the presence of MEG in the MDEA solution increases the reboiler temperature and decreases the acid gas loading (moles of CO_2_ and H_2_S/mole of MDEA) of amine system.

Table 4 shows the simulation results of the gas sweetening unit for five different cases contains 1, 5, 10, 15, 20 and 25 wt% of MEG in the amine solution. H_2_S concentration in sweet gas increased from 1.09 to 3.78 ppm as MEG content increased from 1 to 25% in amine loop. Therefore, the field and simulation results indicated that H_2_S absorption decreased with increasing the MEG concentration in amine loop. But still, MDEA in presence of MEG was kept H_2_S selectivity.

**Table 4:** H_2_S concentration in sweet gas obtained from the simulation for 1 to 25 wt% MEG content in the amine solution

Stream	Composition	Case 1	Case 2	Case 3	Case 4	Case 5	Case 6
Lean amine	MEG (%)	1	5	10	15	20	25
	MDEA (%)	45	45	45	45	45	45
	Water (%)	54	50	45	40	35	30
Sweet gas	H_2_S (ppm)	1.09	1.26	1.74	2.02	3.12	3.78
	CO_2_ (ppm)	14,369.89	14,406.39	14,452.50	14,499.18	14,548.98	14,600.70

The simulation results showed that the energy consumption of regenerator reboiler increases from 39,165,295 (Case 1) to 41,274,795 kJ/h (Case 2). In other equipment, the energy consumption was not changed considerably. Totally, the energy consumption in gas sweetening unit increased 5.4% in the case of 25 wt% MEG in lean amine solution while for 1 wt% MEG, the increase was 0.05%.

### CO_2_ absorption

The CO_2_ absorption in MDEA aqueous solution is carried out via two different reaction mechanisms. When CO_2_ is dissolved in water, the hydrolysis of CO_2_ is occurred to form carbonic acid, which in turn dissociates slowly to bicarbonate. Finally, the bicarbonate undertakes an acid–base reaction with the amine to yield the overall reaction shown through Eqs. (3) to (6):

3 $$CO_{2} + H_{2} O \leftrightarrow H_{2} CO_{3} \left( {Carbonic\,Acid} \right)$$

4 $$H_{2} CO_{3} \leftrightarrow H^{ + } + HCO_{3} \left( {Bicarbonate} \right)$$

5 $$H^{ + } + R_{1} R_{2} R_{3} N \leftrightarrow R_{1} R_{2} R_{3} NH$$

6 $$CO_{2} + H_{2} O + R_{1} R_{2} R_{3} N \leftrightarrow R_{1} R_{2} R_{3} NH^{ + } HCO_{3}$$

**Fig. 6:**
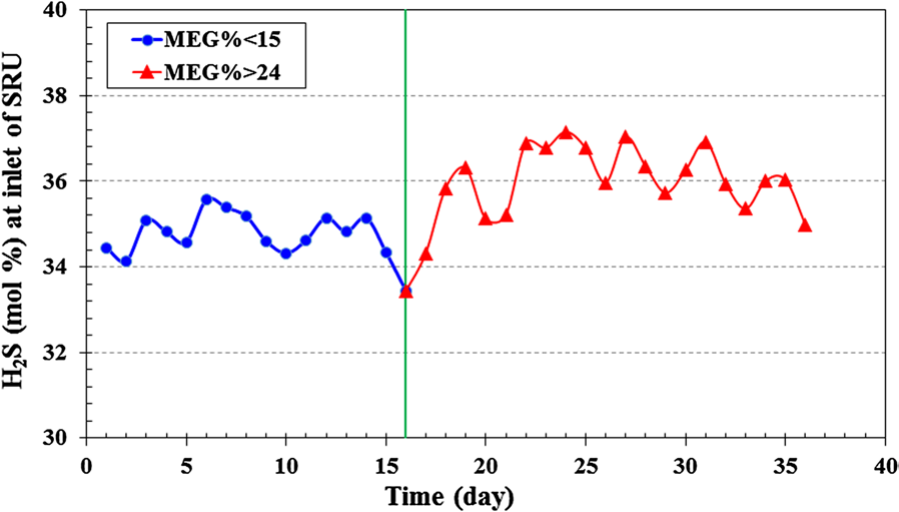
H_2_S concentration in the inlet of the sulfur recovery unit

MDEA reacts with CO_2_ via the slow CO_2_ hydrolysis mechanism [24]. H_2_S reaction with MDEA is fast as compared with the slow CO_2_ reaction with water to form bicarbonate. So, increasing water concentration may lead to an increase in CO_2_ reaction with the amine. With increasing MEG content in amine solution, water content decreases and leads to less CO_2_ absorption from sour gas in the absorber column. It means more CO_2_ loading in rich amine which must proceed in the regenerator. So, CO_2_ loading in the acid gas at the top of the regenerator was increased (Table 4) and consequently, the concentration of H_2_S in SRU feed was increased. The concentration of H_2_S in SRU feed was increased from 35% (MEG% < 15) to 36.5 (MEG% > 24), indicating less CO_2_ absorption in amine absorber was occurred (Fig. 6).

### Corrosion

**Fig. 7:**
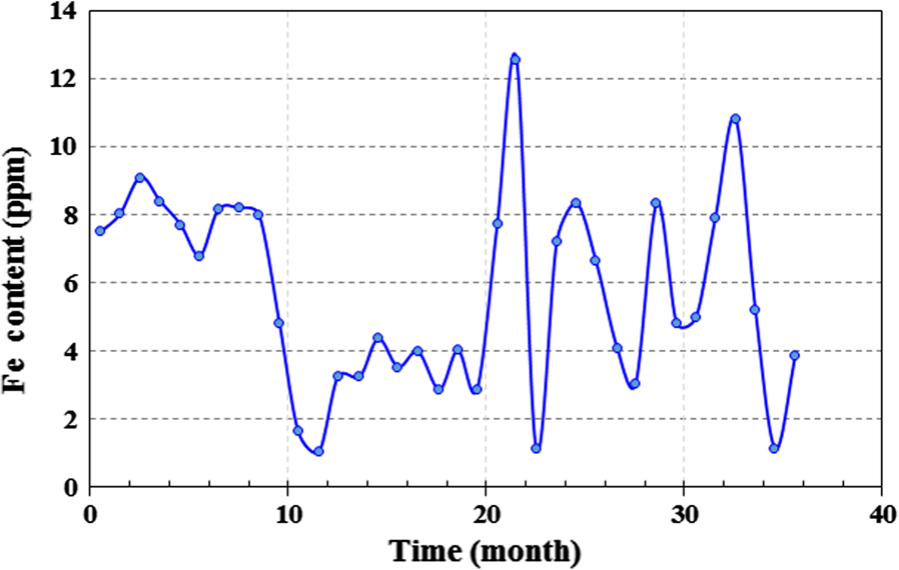
Total Fe content throughout the 36 months in amine gas sweetening loop

**Fig. 8:**
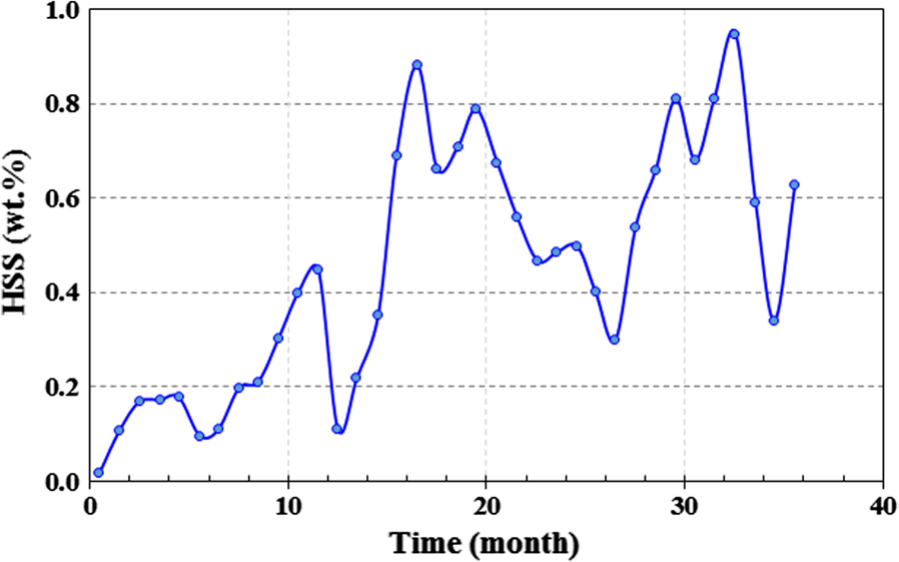
Heat stable salts (HSS) value throughout the 36 months in amine gas sweetening loop

**Fig. 9:**
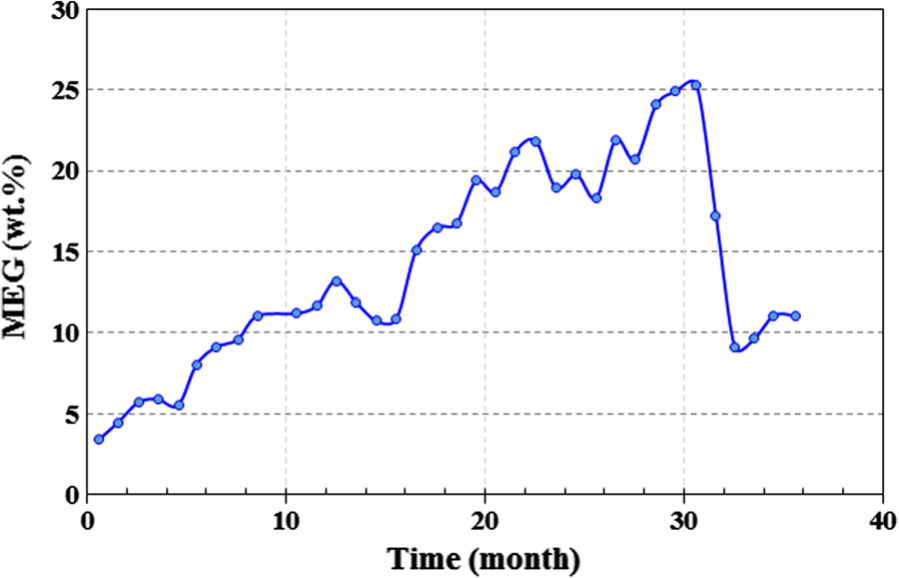
MEG content throughout the 36 months in amine gas sweetening loop

Work equipment in south pars refinery is commonly inspected at suitable intervals (12 months). The inspection of the regenerator and reboiler during 36 months showed severe corrosion in different parts of plants including the vapor line of the reboiler, regenerator tower between chimney tray and tray #7, vapor side of reboiler around the vapor line nozzles, and behind the weir of reboiler. The changes in MEG concentration, HSS, and Fe content in amine loop during 36 months are presented in Figs. 7, 8, 9. As observed, there is a direct relationship between these parameters. Corrosion may cause by HSS through acid evaporation and condensing mechanism in cold spots, as well as, the chelating effect of organic acids and reduction of pH. The high reboiler temperature (131–138 °C) can accelerate the condensation mechanism and acids evaporation. Also, the chemical reaction rate (corrosion) becomes double for every 10 °C rise in reboiler temperature.

**Fig. 10:**
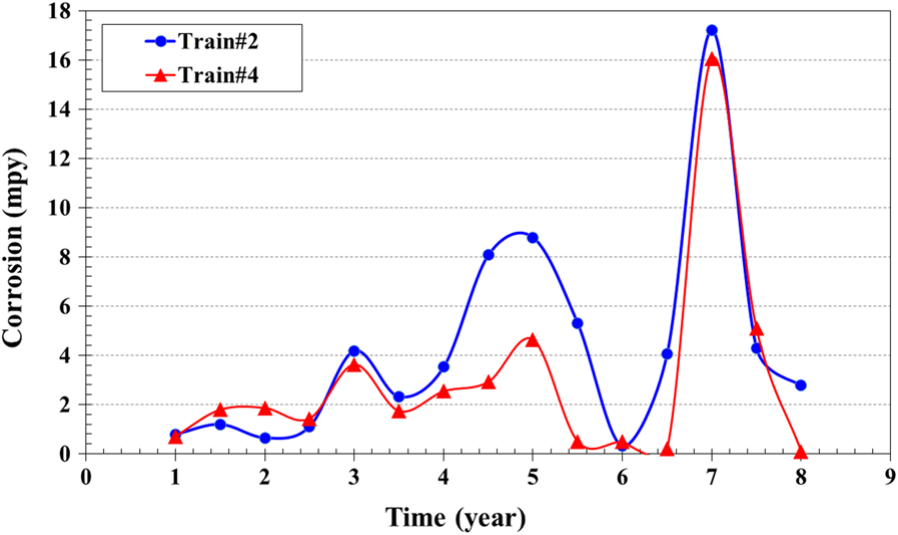
The corrosion rate of regenerator of MDEA unit trains #2 and #4

**Fig. 11:**
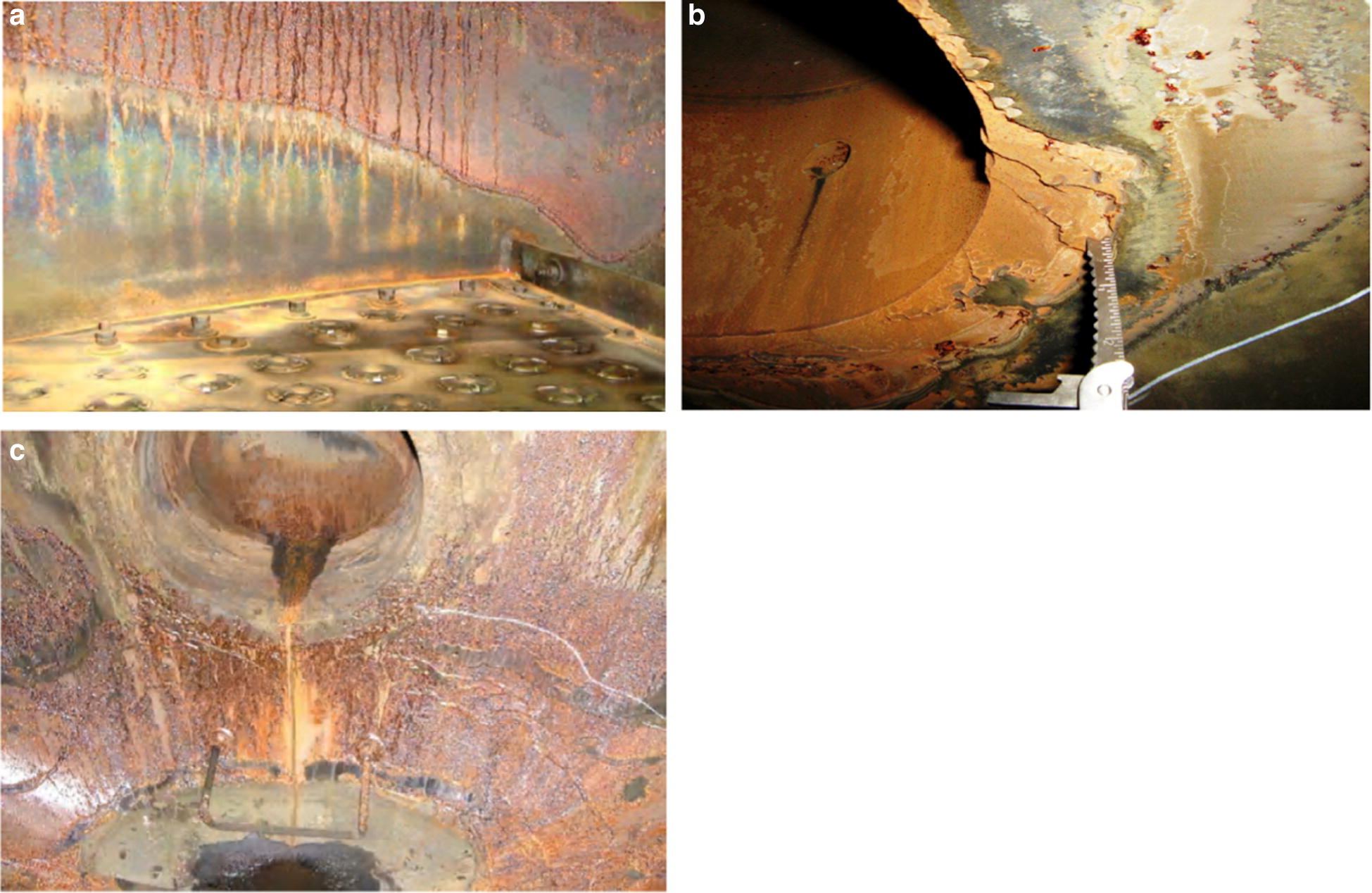
Corrosion **a** in the vapor phase above the normal liquid level through the regenerator tower between chimney tray and tray #7; **b** in vapor side of reboiler around the vapor line nozzles; **c** through the reboiler shell of the regenerator behind the baffle

Under thermal conditions, MEG degrades mainly to glycolic acid with oxalic and partially to formic acid. These degradation products promote corrosion by forming iron complexion. In an amine system, similar to HSS, iron complex enhances the corrosion [8]. The corrosion rate in the gas sweetening unit for 20 and 25% wt% MEG content was 10.5 and 17.2 mpy, respectively (Fig. 10). It is noted that the refinery’s goal is to keep the corrosion rate below 10 mpy. The corrosion rate was less than 10 mpy when MEG content was less than 15%. Figure 11 shows a typical example of corrosion observed in amine gas sweetening unit.

### BTEX and heavy hydrocarbon solubility

Benzene, toluene, ethylbenzene, and xylene (BTEX) are aromatic contaminants that can be permanently poisoned the catalyst of Claus SRU. BTEX can reduce SRU process efficiency and increase the operational cost [25]. The BTEX can be absorbed in the amine solution and removed from the flash drum and if not absorbed they are sent to the SRU. According to the simulation results (Table 5), with increasing 25% MEG content, the solubility of heavy hydrocarbon was increased about 60%. As the amount of BTEX and heavy hydrocarbon were increased, the transferring of these components to the SRU unit was increased. Table 5 shows the content of heavy hydrocarbons in acid gas routed to the SRU. It caused some side effects on SRU performance and leads to sooner catalyst deactivation. A yearly evaluation catalyst was performed in phases 2 and 3. The results showed that the efficiency of catalyst decreased more than expected.

**Table 5:** Composition of acid gas routed to the SRU with lean amine solution containing 1, 5, 10, 15, 20, and 25 wt% MEG content

Composition (mole%)/MEG (wt%)	1%	5%	10%	15%	20%	25%
iC_5_	0.001410	0.001586	0.001868	0.002249	0.002779	0.003540
nC_5_	0.001769	0.001984	0.002326	0.002786	0.003420	0.004325
Benzene	0.067098	0.069330	0.072688	0.076839	0.082082	0.088810
nC_6_	0.000276	0.000311	0.000366	0.000441	0.000544	0.000691
Cyclohexane	0.002220	0.002382	0.002627	0.002936	0.003333	0.003856
Methylcyclopentane	0.000540	0.000574	0.000626	0.000692	0.000776	0.000885
Toluene	0.016273	0.017176	0.018544	0.020261	0.022468	0.025371
Methylcyclohexane	0.000245	0.000266	0.000297	0.000338	0.000390	0.000461
nC_7_	8.20E−05	0.000935	0.000112	0.000138	0.000175	0.000229
nC_8_	3.20E−05	0.000373	4.61E−05	0.000586	0.000769	0.000105
Ortho-xylene	0.018848	0.019907	0.021516	0.023546	0.026170	0.029640
nC_9_	0.000829	0.000964	0.000119	0.000149	0.000193	0.000258
C_10_	0.000221	0.000263	0.000461	0.000435	0.000583	0.000809

### Foaming

Foaming in the amine absorber is a common problem. In an industrial plant, the differential pressure (DP) of the absorber, the flow rate of flash gas (gas exited from the flash drum), and the opening of LV0026 [level valve of the bottom of sweet gas Knock-Out (K.O)] are signs of foaming. Parameters such as sour gas inlet temperature, bottom level of absorber, amine flow rate and temperature, gas flow, antifoam concentration, homogeneity and flow rate, lifetime of filters, total suspended solids (TSS) of amine, and lean amine quality have significant effects on foaming formation.

**Fig. 12:**
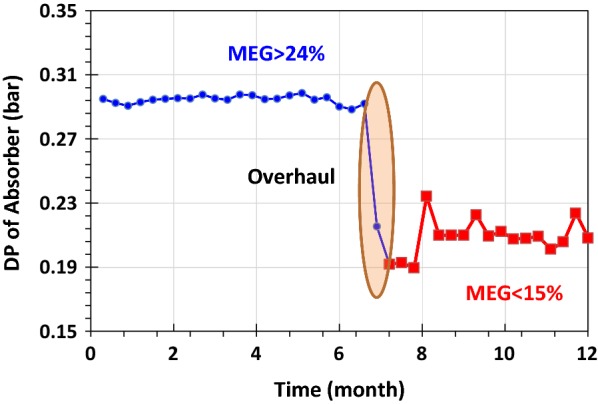
Differential pressure of amine absorber, overhaul: scheduled shutdown maintenance

Amine absorber is equipped with DP cells to monitor system abnormalities. As such observed in this plant (Fig. 12), DP of the absorber can be increased up to 0.3 bar. When foaming is formed in the absorber, the foam height increases with time, and subsequently, the void volume inside the column reduces, leading to higher pressure drop.

After removing MEG from lean amine, the opening of LV0026 shows amine carryover and DP of absorber were decreased from 0.3 to 0.2 bar (Fig. 12). These signs showed foaming are reduced in amine loop and the used amine has more TSS in compare to the fresh amine.

**Fig. 13:**
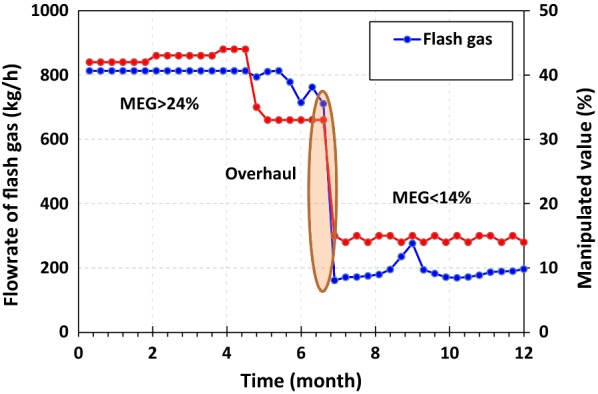
Flash gas from the flash drum and LV0036 opening overhaul: scheduled shutdown maintenance

When there is severe foaming in the absorber, amine carryover from the absorber to sweet gas K.O drum. While other effective parameters were in relatively constant conditions, flash gas and the opening of LV0026 were in a direct relationship with MEG concentration (Fig. 13). The operation signs clearly confirmed excessive foaming with 25 wt% MEG concentration in amine loop.

### MDEA contaminant analysis

The degradation products, HSS, metals and other contaminants of amine in presence of 25% MEG were analyzed and the results are reported in Table 6. Furthermore, in this paper, for the first time, all necessary information for academic and industrial users, according to the literatures [24, 26–32] and our industrial experiences, were brought out in a table (Table 5) which contains the allowable limit, source and effects of each contaminant in amine loop and the pros and cons of various operational conditions in amine gas sweetening processes. This information leads users to investigate their own unit circumstance. However, to more evaluation, the composition of used amine was analyzed. The results obtained here showed that the composition of all components are in the allowable range but the composition of acetate in all gas treating units is more than allowable limit (1000 ppm), indicating MEG presence in amine loop.

**Table 6:** The amine analyses results, allowable limit, source, and effects of contaminant

Component	SPEC	Train #2	Train #1	Train #3	Train #4	Source	*Notes*
H_2_O (wt%)		34.6	48.4	39.4	41.3		
Total amine content (wt%)		43.5	43.1	45.9	43.5		
MDEA (wt%)	50	43.4	32.8	33.3	32.9		
MEA (wt%)			0.1	0.1	0.1	Degradation in the presence of oxygen	Can be turned to *N*-(2-hydroxyethyl) ethylenediamine (HEED) It is non-corrosive It promotes thermal degradation of MDEA in presence of oxygen
DEA (wt%)	5000 ppm		0.1	0.1	0.1	In the presence of oxygen at a temperature above 82° C	DEA is formed carbamic acid with CO_2_, this acid can be turned to the *n*,*n*,*n*-tris-(2-hydroxyethyl) ethylenediamine (THEED). THEED corrosion rate is 6 times higher than DEA
MEG (wt%)		19.8	8.3	15	14		
Iron (ppmv)	20 mg/l	< 1	1	3	1	Production of corrosion or erosion	
Nickel (ppmv)		2.8	1	1	1	Production of stainless steel corrosion	Must be monitored and checked by corrosion coupon
Chromium (ppmv)		< 1	1	1	1	Production of stainless steel corrosion	Must be monitored and checked by corrosion coupon
CO_2_ (mol CO_2_/mol MDEA)	0.005	< 1				CO_2_ in regenerated amine	Helped to the corrosion with formation of HSAS
H_2_S (ppmv)		< 1					
Acetate (ppmv)	1000	3584	1250	3350	2200	Combination of amine, glycol with oxygen	
Formate (ppmv)	500	108	90	140	120	Reaction of amine with oxygen at temperature above 121 °C	For 2000 ppm formate, severe corrosion occurs especially in the top of the regenerator
Chloride (ppmv)	200	52	300	40	20	In make-up water and in feed gas	With amine formed amine chloride Increases the pitting corrosion Leads to the corrosion and erosion of stainless steel and total corrosion of carbon steel
Sulfate (ppmv)	500	80	30	130	75	Oxygen of make-up water is reacted with H_2_S	Increases the rate of corrosion Can be formed Bicine
Oxalate (ppmv)	250	< 1	15	25	25	Reaction of amine with oxygen at temperature above 81 °C	Chelating agent Increases the corrosion
Thiosulfate (ppmv)	10,000	< 1	15	40	10	Entering oxygen to the system	Purging the water of reflux drum can reduce it
Thiocyanate (ppmv)	10,000	< 1	10	10	10	In the feed gas H_2_S + O_2_ + HCN	Non corrosive
Nitrate (ppmv)		< 1					
Phosphate (ppmv)		< 1					
Glycolate (ppmv)	500	270	300	430	250	Reaction with oxygen in temperature above 82 °C	Causes the corrosion
Butyrate (ppmv)		< 1	10	10	10		
Sodium (ppmv)	200	< 1	20	20	20	Water make up	
Potassium (ppmv)		< 1	10	20	10	Water make up	
Ammonium (ppmv)	10,000	< 1				Amine thermal and oxygen degradation. Side production of cyanide with water	If ammonium is condensed, it absorbed CO_2_, formed carbonate ammonium or bio carbonate and block the condenser path It can absorb H_2_S and formed biosulphide that it is corrosive
Magnesium (ppmv)		< 1	1	1	1		
Calcium (ppmv)		< 1					
pH	> 10	10.3	9.9	9.7	9.8		
Total solid content (wt%)	10 ppm	0.013	3.7	6.7	6.7	Weak in primary separation, corrosion from the filters	TSS must be less than 100 ppm
Average particle size (µm)		6.5					The average particle size shall be less than 5 μm to prevent foaming
Silicon (ppmw)	25		25	25	30	Antifoam	It absorbed in the carbon filter and covers the cartridge filter
Amino acid (ppm)			10	10	10		
Bicinne	250					MDEA covert to TEA. TEA reacts with oxygen to form bicine Cyanide + formaldehyde	Severe corrosion especially in reboiler Chelating agent If bicine is more than 250 ppm, corrosion more than 10 mpy is expected for carbon steel Can be removed by vacuum distillation and ion exchange
Manganese	0.5 PPMV					Carbon steel corroded	
MMEA						In the presence of oxidant and acids, MDEA converts to MMEA at high temperature	Can be converted to the DMHEED Non-corrosive Can be made situation with potential for corrosion Can be removed by vacuum distillation
Acid acetic						Temperature more than 121 °C and presence of oxygen	Water washing before absorber can be reduced it
Oxygen						Fitting and metering in wellhead equipment, lines are corroded or amine tank if has not nitrogen as inert gas	In presence of oxygen, MDEA, after a while, converts to the DEA For less amount of oxygen, oxygen scavenger such as hydrazine, amine hydroxyl can be used Nitrogen blanketing in amine tank Oxygen solubility in amine is 2 to 10 ppmv 100 ppmv of oxygen in feed gas can produce high amount of HSS
HSS	0.5 to 1.0 wt%					When amine react with acids stronger than H_2_S and CO_2_	Increases foaming, viscosity and mass transfer, decreases capacity of acid gas absorption
CO_2_/H_2_S						For ratio less than 19, total acid gas in amine increases relatively because of protection layer of FeS	
Color						Dark coffee from corrosion Dark brown from thermal destroyed	When the amine is brown, after passing of filter paper, the color is changed, the source is corrosion otherwise the source is amine thermal degradation
Foam tendency	Nil to 30 s					Hydrocarbon and solid particle	

### Operational remedies

There are numerous operational problems in the gas sweetening unit, especially excessive corrosion. In order to overcome these challenges, some techniques were carried out as follows:

- Dropping the bottom temperature of amine regenerator:
  In this technique, the temperature and pressure at the top of regenerator must be reduced. The temperature has a positive effect but the pressure has not considerable effect. Moreover, rich amine existed from flash drum is entered to the amine/amine exchanger and then routed to the regenerator. If the efficiency of amine/amine exchanger increases, the temperature of amine fed to the regenerator will be increased and consequently less steam is needed in the reboiler and the bottom temperature of regenerator can be kept in lower temperature. But from the economical point of view, this technique was not possible.
- Applying a coating of Ceramium on the bottom of the regenerator and around the nozzles of reboiler.
- Applying proper insulation in the corroded area over the vapor line to prevent condensation.
- Changing the material of the vapor line of reboiler from carbon steel to stainless steel—grade 316 (SS316).
- Using partially refreshment of fresh MDEA (0.5 to 5.0%).

**Fig. 14:**
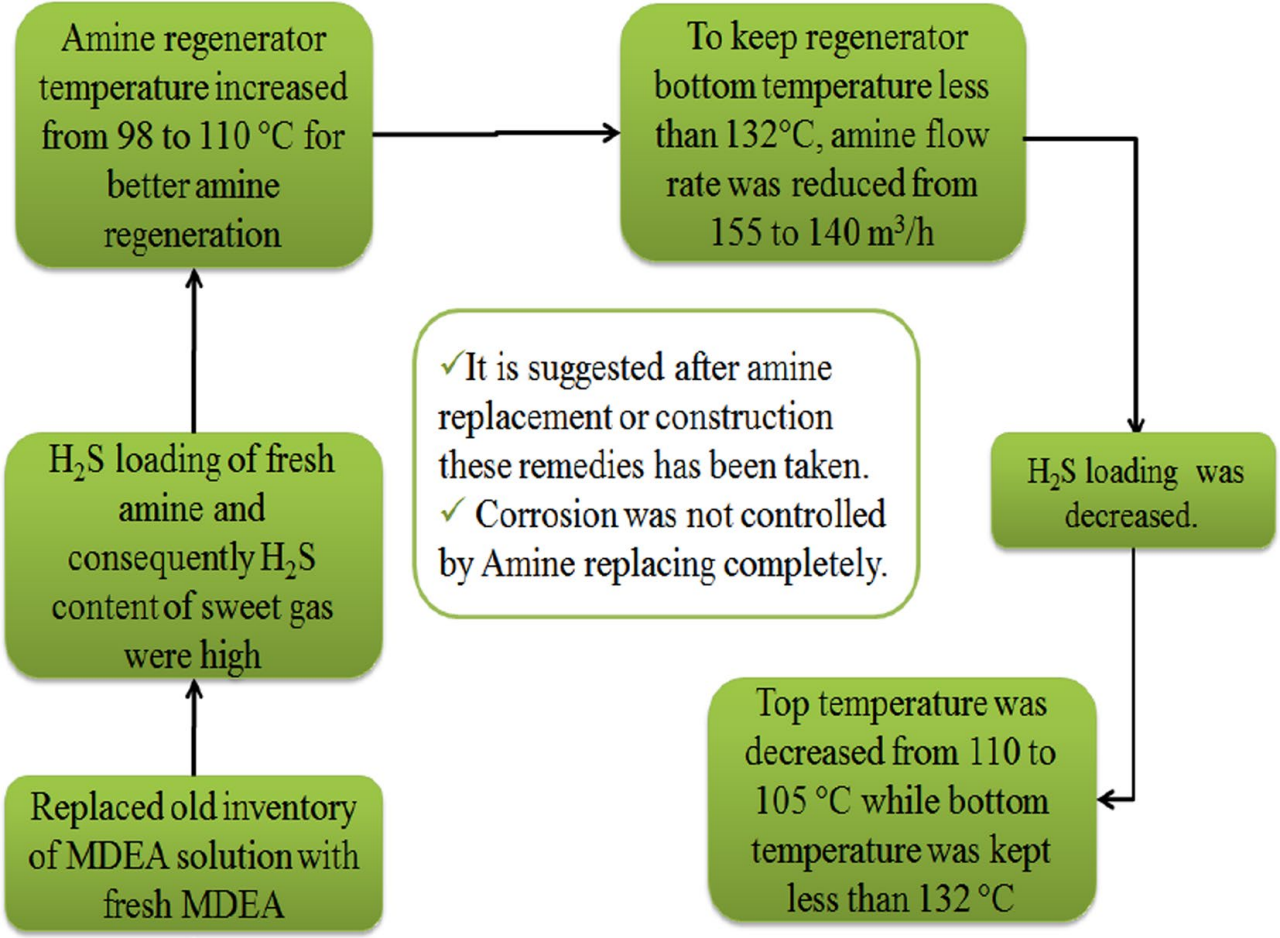
Operational remedies after total amine replacement

These techniques were effective but not enough. Since there is not any facility for amine purification, it was decided to replace used MDEA with a fresh one and the steps of this operational remedy are pictured in Fig. 14 [33].

After using fresh amine, the H_2_S content in both fresh amine and consequently in sweet gas were high, indicating acid assisted regeneration phenomena [33]. To reduce H_2_S loading in amine solution and better amine regeneration, the temperature of amine regenerator was increased from 98 to 110 °C and the bottom temperature of regenerator was increased according to the temperature at top of the regenerator. It must be emphasized to this point that high bottom temperature can cause amine degradation. To keep regenerator bottom temperature less than 132 °C, the amine flow rate was reduced from 155 to 140 m^3^/h. Lower amine flow rate increases MDEA residence time in the regeneration section and as a result, H_2_S loading decreases. Therefore, the top temperature of regenerator was decreased from 110 to 105 °C while the bottom temperature was kept less than 132 °C. Since the fresh amine creates some problems in the amine gas sweetening unit, refreshment was partially carried out in order to keep MEG content less than 10 wt%. With results of this experience, it is suggested a few used-amine is added to the fresh amine after the construction of the amine gas sweetening unit.

These solutions were used to reduce the side–effects of MEG. Therefore, it must be found an operational remedy to avoid entering MEG to amine plant. To achieve this purpose and regarding the design, the sweet gas is routed to the gas dehydration unit and is then entered to the K.O drum (105-D-X01, where X = 1, 2, 3, and 4) of dew pointing unit. Bottom of this drum is returned to the amine flash drum. Based on the simulation results, there is a considerable amount of MEG (between 0.5 and 4.0 wt%) in the bottom stream of the K.O drum. Table 7 shows the actual and simulated data of MEG% in this stream.

**Table 7:** Comparing actual and simulated data of MEG wt% in the bottom of 105-D-201

MEG% in lean amine	MEG wt% in bottom of 105-D-201	
	Simulated	Actual
0	0.00	0.00
5	0.58	0.51
10	1.22	1.22
15	1.94	1.95
20	2.76	2.78
25	3.71	3.73

Therefore, it was decided that this line be routed to the stabilization condensation unit in gas train #2 (second train) instead of routing to the amine flash drum. The simulation of this plant also indicated that the equilibrium amount of MEG in lean amine is 14 wt%. When the bottom of the K.O drum is not routed to the flash drum and the concentration of MEG in amine loop is more than 14 wt%, the amount of MEG in amine loop decreases. It was found that when the MEG concentration in amine loop is less than 14 wt%, this remedy cannot reduce the MEG loading in amine loop. The MEG loading in lean amine after applying this change is shown in Table 8.

**Table 8:** MEG loading in lean amine after routed to the bottom of 105-D-201 in the condensation unit instead of routing to the amine flash drum

Month	MEG%	Month	MEG%	Month	MEG%
1	26.00	13	10.09	25	11.44
2	24.20	14	11.33	26	11.51
3	23.00	15	11.48	27	11.87
4	19.50	16	10.62	28	10.80
5	18.80	17	10.21	29	11.52
6	13.60	18	10.10	30	11.14
7	15.00	19	13.10	31	12.44
8	12.20	20	8.50	32	10.32
9	13.30	21	9.28	33	10.64
10	11.15	22	8.49	34	11.00
11	12.01	23	9.48	35	14.11
12	12.19	24	9.80	36	14.10

Moreover, increasing amine loss and consequently amine make-up may reduce MEG content in the gas sweetening plant. Hence, the amount of amine make-up was monitored to find whether MEG content in the gas sweetening plant is actually reduced or not. Therefore, the MDEA make-up in different gas treating units was compared (Table 9) indicating normal status in all trains.

**Table 9:** MDEA make-up in gas sweetening unit train #1 to #4

Train #	1	2	3	4
MDEA Make-Up (m^3^)	11.26	40.79	138.65	141.60

In addition, with consideration of operational parameters, this line (bottom of 105-D-X01 routed to the condensation unit) must be checked from the corrosion point of view. Therefore, corrosion coupon was installed in the route. After 6 months, the installed corrosion coupons showed corrosion rate less than 1 mpy (allowable limit of NACE standard RP 0775). Consequently, by applying the proposed operational remedies, the MEG loading in amine loop has kept less than 15 wt% for 3 years.

## Conclusions

In this paper, the presence of MEG in MDEA loop in phases 2 and 3 of south pars gas field was evaluated. Summary of the findings are presented as follows:

- Introducing 25 wt% MEG in amine loop decreases H_2_S and CO_2_ absorption from sour gas.
- Introducing 25 wt% MEG, the regenerator bottom temperature was increased from 129 to 135 °C and consequently, energy consumption of the sweetening unit was increased 5.4%.
- Because of less CO_2_ absorption in absorber column, H_2_S concentration in inlet SRU was increased. Also, the solubility of BTEX and heavy hydrocarbon in amine solution was increased, which leads to transferring BTEX to SRU and finally sooner catalyst deactivation.
- Foaming problems were increased.
- Severe corrosion was observed in some parts of the regeneration section. Since approximately all the contaminations of amine were in the allowable limit, the reason for the corrosion just can be related to the MEG presence and higher temperature of the regeneration section.
- Total and/or partial refreshment of fresh MDEA was used in gas sweetening unit to reduce MEG content. Furthermore, some techniques (install insulation, coating, etc.) in point of prevention of corrosion were carried out in regenerator tower.
- Bottom of the inlet K.O drum of the dew pointing unit (105-D-X01) was routed to the stabilization unit instead of routing to the amine flash drum. Hence, the MEG presence in lean amine was kept less than 15 wt% until now.
- The value, allowable limit, source and effects of each contaminant and the pros and cons of operational conditions in amine gas sweetening were illustrated.
- It is recommended to consider the effects of MEG in amine loop in the design of gas sweetening unit when glycol exists in the offshore.
